# Neurological manifestations in mild and moderate cases of COVID-19

**DOI:** 10.1186/s41983-021-00363-8

**Published:** 2021-08-04

**Authors:** Ghada Saed Abdel Azim, Marwa Abdellah Osman

**Affiliations:** Neurology Department, Faculty of Medicine-for Girls, Al Azhar University, Cairo, Egypt

**Keywords:** Neurological manifestations, Home-isolated patients, COVID-19

## Abstract

**Background:**

The coronavirus disease due to SARS COVID-2 emerged from Wuhan city in China in December 2019 and rapidly spread to more than 200 countries all over the world as a global health pandemic. Its primary presentation is respiratory and cardiac. However, some neurological manifestations are also reported. We tried to explore the reported neurological manifestations in a group of non-hospitalized mild and moderate COVID-19 patients. We contacted 107 patients via phone calls and e-mail messages, within 10 days of clinical presentation. The collected data regarded the neurological and non-neurological symptoms of the disease using a questionnaire that collected medical information of each patient.

**Results:**

It is found that 100% of patients have been reported with at least one neurological symptom during the first 10 days of COVID-19 presentation. The most common were headache which recorded 72% of the total. Then anosmia–dysgeusia which reached 52%, then myalgia with 44%, fatigue with 33% and dizziness with 32%. While the less common was numbness, migraine, loss of concentration, and seizures.

**Conclusion:**

There are many neurological manifestations found to be very common in COVID-19 patients even in mild cases, which when added to the increasing reports of serious cases of Guillain–Barre syndrome, acute necrotizing encephalopathy, myelitis, stroke, and encephalitis in COVID-19 patients support CNS invasion of the virus and assures the importance of neurological assessment of COVID-19 patients both in the acute phase of infection and after recovery for potential neurological sequelae.

## Background

The coronavirus (COVID-19) pandemic originated in Wuhan city in China in December 2019. Most patients infected by the virus have presented with a mild clinical course, starting with fever and dry cough, progressing to mild or moderate respiratory disease. However, more serious complications of the infection, such as acute respiratory distress syndrome, acute heart failure, and acute kidney injury have been reported in COVID-19 patients, particularly among older age patients or patients with underlying comorbidities [1]. Neurological manifestations are not uncommon, and they were reported in COVID-19 patients in the literature early in the pandemic. Neurological complications were reported as a part of previous pandemics with respiratory organisms such as H1N1 influenza, MERS and SARS. Those complications could occur in the acute phase of the illness, due to viral invasion of the CNS or due to parainfectious cytokine storm or post-infectious, due to cellular immune or antibody-mediated immune phenomenon such as Guillain–Barre syndrome. However, the percentage of patients who have these complications may be small and they are often the most severely affected, needing intensive care admission and resulting in poor outcome [2].

The previous reports on neurological complications of COVID-19 are restricted to few cases or case series of hospitalized patients presenting with more severe illness. But, how common are neurological symptoms in mild non-hospitalized cases of COVID-19, and whether it affects the central or the peripheral nervous system, and are they correlated with symptoms of other affected organs?

To answer these questions, we collected data of a group of non-hospitalized mild-to-moderate cases of COVID-19 through phone calls, as they were isolated for diagnosis of COVID-19.

## Methods

We contacted 107 patients with the following inclusion criteria: symptomatic cases with leucopenia and lymphopenia and radiological evidence of COVID-19 pneumonia, symptomatic cases with confirmed COVID-19 infection by PCR in a nasopharyngeal swab and they did not require admission to the hospital. We contacted them within 10 days of clinical presentation, during the period of home isolation. The exclusion criteria were the inability to communicate due to speech impairment or cognitive disability.

The study was conducted in Beni Suef City, Egypt, during the period from the 15th of June to the 1st of August 2020.

An inquiry form to collect the symptomatology of the nervous system in a group of home-isolated COVID-19 patients was used. The form included the neurological symptoms which are mentioned in the previous literature of COVID-19. We also gathered information about the medical history of the patients. The collected data included, but not limited to, the following: age, sex, the onset of the disease, method, and place (health care facility or institution) of diagnosis. Also, history of diabetes, hypertension, cardiac disease or respiratory disease was obtained. In addition, history of headache, epilepsy, stroke, or dementia was collected. Then, neurological manifestations at the onset and after diagnosis of COVID-19: headache (and its criteria and type), anosmia–dysgeusia, muscle pain, dizziness, encephalopathy (disturbed attention, delirium, or confusion), seizures, numbness, fatigue were registered. In addition, non-neurological symptoms at presentation, fever at the onset, respiratory symptoms, or gastrointestinal symptoms were collected.

Results of laboratory investigations when available were obtained by e-mail such as total and differential leukocyte count, serum ferritin, D-dimer, C-reactive protein and reports of CT chest imaging.

Verbal informed consent was taken from all patients as they were in the period of home isolation subsequent to diagnosis of COVID-19 infection.

Statistical methods: the data were recorded and analyzed by Statistical Package for Social Sciences, version 20.0 (SPSS Inc., Chicago, Illinois, USA). The description of the variables was carried out by using frequency tables, means and standard deviations (SD), and linear correlation coefficient.

## Results

**Fig. 1:**
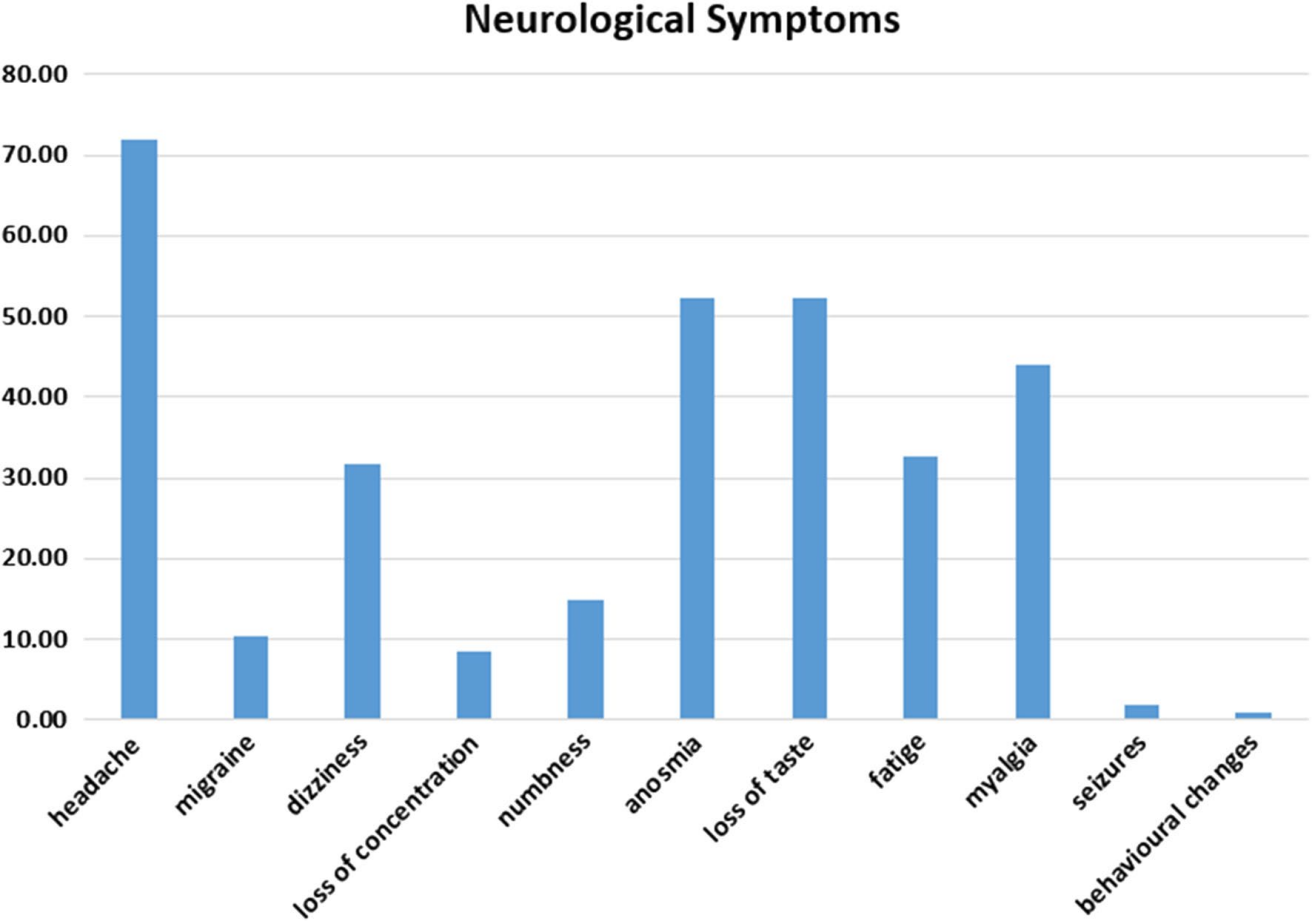
Neurological manifestations associated with COVID-19

**Fig. 2:**
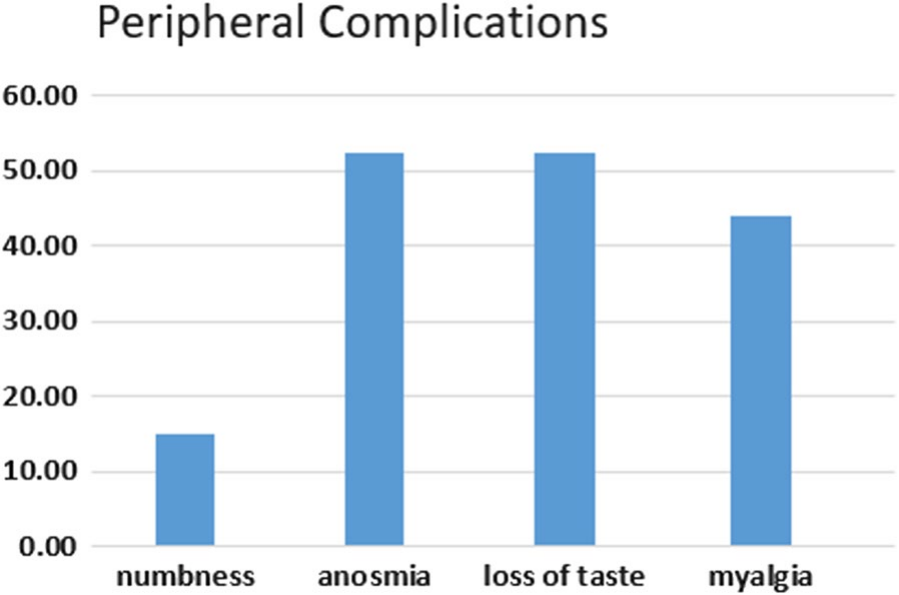
Peripheral nervous system manifestations

**Fig. 3:**
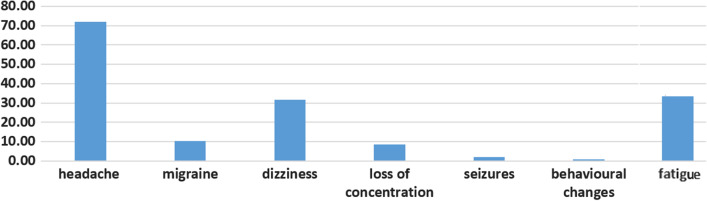
Central nervous system manifestations

One hundred and seven patients participated in the study. Fifty-one of them are males (47.6%) and 56 of them are females (52.4%). The mean age, sex, and neurological complications are listed in Table 1, while Table 2 tabulates the neurological complications associated with COVID-19 and depicted in Fig. 1 as bar charts. Also, Fig. 2 charts the peripheral nervous system complications ratios in bars representation. The central nervous system complications are represented in Fig. 3 in bar chart.

**Table 1:** Demographic data and distribution of neurological symptoms

Number of patients	107	
Age (mean ± SD)	41.23 ± 13.94	
Sex	Male	Female
N (%)	51 (47.6%)	56 (52.4%)
Percent of neurological complications	Central	Peripheral
	84 (78.5%)	89 (83.17%)

**Table 2:** Neurological manifestations associated with COVID-19

Manifestation	Percentage (%)
Headache	71.96
Anosmia	52.34
Dysgeusia	52.34
Myalgia	43.93
Fatigue	32.71
Dizziness	31.78
Numbness	14.95
Migraine	10.28
Loss of concentration	8.41
Seizures	1.87
Behavioral changes	0.93

## Discussion

While coronavirus 2 (SARS-CoV-2) epidemic spreads, it appears that COVID-19 infection is not restricted to the respiratory system; other organs can be affected too. Specifically as more as the disease spreads, neurological complications in patients with COVID-19 are reported more frequently in the literature [3]. In this study, we investigate the presence of neurological manifestations in 107 home-isolated COVID-19 patients. We noticed that 100% of patients present at least one neurological manifestation. So, this was consistent with Garcia-Monco and colleagues who reported that 88% of patients (not required ICU admission) had at least one neurological manifestation [4]. However, this is counterpointed to the study of Mao and colleagues from Wuhan, China who reported that neurological manifestations affect 36% of the patients [5]. In addition, this was contradictory to Pinna and colleagues who described neurological symptoms in 50 out of 650 patients (7.7%) hospitalized with COVID-19 in Chicago, Illinois [6], and Karadaş and colleagues who identified neurological symptoms in 83 out of 239 patients in Ankara, Turkey [7] .The difference in results could be explained by the differences in patients’ characteristics as those studies were carried out on hospitalized patients with severe COVID-19 infection. Moreover, the information in those studies was collected retrospectively from electronic medical records. Besides, neurologic manifestations might not be noticed if they were too mild, such as with taste impairment and smell impairment.

It is noticed that headache was a common symptom (77%) of patients followed by anosmia and dysgeusia (52%) of patients. This is in accordance with Garcia-Monco and colleagues who reported that headache and anosmia–dysgeusia were of the most prevalent neurological symptoms with a ratio (44%) and were more prevalent in mild cases of COVID-19 [4]. Headache presented in 13.1% of hospitalized severe cases of COVID-19 in comparison to mild cases (17% vs. 10.3% in less severe cases) [5].

Headache was characterized by moderate-intensity, dull aching, persistent resembling tension-type headache in most of the patients (67% vs. 10% migraine-like), and two patients with occipital headache. In general, the patients in our study had no history of previous primary headaches, thus, the headache could be caused by the infection and not deterioration of pre-existing primary headache. The high frequency of headaches in mild and moderate cases of our study could reflect the overwhelming of severe respiratory symptoms in severe cases of COVID-19 over the neurological symptoms which reflected by lower rate of headache in initial reports of severe respiratory COVID-19 cases. Conversely, Yan and colleagues revealed that anosmia and dysgeusia were more prevalent in the COVID-19-positive cases in comparison to the negative cases (smell loss: 68% versus 16% and taste loss 71% versus 17%), using an internet-based cross-sectional survey. The majority of the patients in that study were ambulatory and did not require hospitalization. They supposed that in mild ambulatory COVID-19 patients virus spreads via the nasal route as compared to the severely affected patients, in whom the spread is most likely pulmonary [8]. On the other hand, Mao and colleagues in their study cohort of 214 Chinese patients demonstrated impairment of taste in 12(5.6%) and impairment of smell in 11(5.1%) patients [5].

The main genes, which included in COVID-19 entrance, are ACE2 and TMPRSS2. They are expressed in both the nasal epithelium and support cells of olfactory epithelium. But not in olfactory sensory neurons, signifying a non-neural mechanism for anosmia, which seems to oppose the assumption that the virus directly invades the nervous system [9].

Myalgia was present in 44% of our patients as it was reported in the study of Garcia-Monco and colleagues, who concluded that it was more in women and was not associated with inflammatory markers or serum CK values [4]. Unfortunately, in this study, we could not order the CK serum levels of our home-isolated patients.

Mao and colleagues stated that skeletal muscle injury was present in (19.3%) of patients especially the severely ill and in (4.8%) of patients in the non-severe group. But, it was indistinct whether the cause is through effect of the virus on muscle tissue, or the immune response mediated by infection which resulting in increased cytokines in the serum leading to skeletal muscle injury [5].

In accordance to the study of Garcia-Monaco and colleagues, 31% of patients presented with dizziness. Dizziness was not associated with vertigo and it seems to be a non-specific symptom in the context of systemic infection not due to true vestibular dysfunction. The incidence of dizziness ranges from three to 12.1% in different reports of COVID-19 presentation [10, 11].

Fatigue is a frequent complaint in patients presenting with symptomatic COVID-19 infection. It was listed as a presenting symptom in 44–69.6% of infected individuals in the early reports of COVID-19 clinical characteristics. Additional studies were followed by meta-analysis, with 34–46% of those patients who suffer from fatigue at presentation, while it was present in 32.71% of our patients [12].

Less common in this series was paresthesia (14%) of patients. Paresthesia was described as mild tingling and numbness in the hands and legs. We thought it could be due to anxiety or maybe a side effect of medication (chloroquine and hydroxychloroquine). However, there were reports of neuropathic pain in severely affected cases in the retrospective study by Mao and colleagues [5]. Moreover, three reports of six patients with COVID-19-related neuropathy were published. The authors suggested that the type of neuropathy that affects their patients was not due to Guillain–Barre syndrome (GBS) [13–15]. Chaumont and colleagues reported four patients (age ranged from 52 to 72 years, all males) who presented with neurological symptoms accompanied by quadriparesis. The data indicate that 3 patients had demyelinating polyradiculoneuropathy; however, the fourth could suffer axonal neuropathy. The neuropathy was asymmetrical in one patient [15]. The mechanism of peripheral neuropathy caused by COVID-19 may be autoimmune as in GBS, or due to the direct cytotoxic effect of the virus on peripheral nerves [16].

Among the less frequent manifestations in our series were loss of concentration, seizures, and behavioral changes. However, they were transient and mostly associated with the presence of fever. In accordance with these results, Garcia-Monco and colleagues reported that seizures were present in 2% of patients in their series and have been reported in a few patients to date [4]. At this time, it is not clear whether the seizures were coincidental or due to SARS-CoV-2 viral effects or caused by the medications used for treatment.

Neurological involvement in COVID-19 may be due to direct viral injury to the nervous system or through indirect mechanisms. Viruses can enter the CNS through hematogenous spreading and neuronal retrograde dissemination. In the hematogenous route, the viruses disseminate all over the body via the blood stream and then go into the brain by crossing the blood–brain barrier. While in retrograde viral spreading, the virus infects the peripheral neurons and transported via the cells to invade the CNS [17].

Although the micro-invasiveness of SARS-CoV-2 has not yet been confirmed, multiple pieces of evidence suggest that other human coronaviruses can use both the blood stream and dissemination through neurons to infiltrate the CNS [18]. Once infecting the lungs, coronaviruses can penetrate the epithelial barrier, get into the blood stream and enter the CNS by invading the endothelial cells of the BBB, or infecting the epithelial cells lining the choroid plexus of the blood–CSF barrier. Furthermore, it can invade leukocytes that are monocytes, granulocytes, and lymphocytes. After being activated by infection, these leukocytes spread to other tissues and cross the BBB. After entering into nervous system, leukocytes produce pro-inflammatory cytokines such as TNF which results in injury of both oligodendrocytes and neurons [19].

Moreover, the spike protein on the surface of SARS-CoV-2 bonded to the ACE2 receptor on the host cell is very important to infection. Furthermore, ACE2 present on the neurons, astrocytes, and oligodendrocytes. In addition, they are found in the ventricles, substantia nigra, posterior cingulate cortex, middle temporal gyrus and olfactory bulb. Due to the extensive expression of the ACE2 receptor in the CNS it could be assumed that SARS-CoV-2 could infect neurons and the glia all over the CNS [20].

Our study has the following limitations; first, the recorded symptoms were based on subjective descriptions provided by the patients, who were in isolation. Second, we could not perform a neurological examination, or advanced neuroimaging such as MRI, or diagnostic procedures as electromyography during the outbreak. Third, the neurological symptoms collected in the first few days of presentation, we contacted every patient once or two times mostly within the first week of home isolation and unfortunately, we did not arrange for repeating contact in regular times and we could not tabulate the time of contact of every patient. However, long-term neurological effects should not be overlooked.

## Conclusion

The study reflected a significant rate of neurological involvement in non-hospitalized individuals with COVID-19 infection in the community, which when added to the increasing reports of serious cases of Guillain–Barre syndrome, acute necrotizing encephalopathy, myelitis, stroke, and encephalitis in COVID-19 patients support CNS invasion of the virus and assures the importance of neurological assessment of COVID-19 patients both during the acute phase of illness and after recovery for potential neurological sequelae.

## Data Availability

The datasets used during the current study are available from the corresponding author on reasonable request.

## Abbreviations

BBB: The blood–brain barrier
COVID-19: Coronavirus disease of 2019
SARS: Severe acute respiratory syndrome
SARS-CoV-2: Severe acute respiratory syndrome corona virus type 2
MERS: Middle East respiratory syndrome-related corona virus
H1N1: Hemagglutinin 1 Neuraminidase 1 (Influenza A virus subtype H1N1)
ACE2: Angiotensin-converting enzyme 2
TMPRSS2: Transmembrane protease serine 2
CK: Creatine kinase
GBS: Guillain–Barre syndrome
CNS: Central nervous system
MRI: Magnetic resonance imaging
TNF: Tumor necrosis factor
